# Supplementary material for: When safety becomes the priority: defensive nursing practice and its associated factors among nurses in Egypt: a cross-sectional study
Source: PeerJ. 2025 Feb 25;13:e19005. doi: 10.7717/peerj.19005 (PMC11869891; doi:10.7717/peerj.19005)
Supplement: Supplemental Information 2 [file peerj-13-19005-s002.docx]

**Characteristics of studied nurses:**

| Items |
| --- |
| **Age:** |
| 20 - <30  30 - <40  40 – 50 |
| **Gender:** |
| Female  Male |
| **Marital Status:** |
| Single  Married  Divorced  Widowed |
| **Qualifications:** |
| Nursing Diploma  Nursing institute  Bachelor’s degree  Postgraduate |
| **Years of working experience:** |
| 1 - <10  10 - <20  20 – 30 |
|  |
| **Specialty (Department):** |
| ICU  Ward  Emergency  Operation  Psychiatry |
| **Experienced physical violence in the workplace:**  Yes  No |
| **Threatened by patients or their companions that you might face legal consequences:**  Yes  No |
| **Experienced legal consequences due to circumstances related to nursing practice:**  Yes  No |
| **Risk of malpractice lawsuit at work:**  Profoundly high  High  Not at all |

**Practices of defensive nursing among studied nurses:**

| **The practices of defensive nursing** | Never | Sometimes | Always |
| --- | --- | --- | --- |
| Carry out interventions or procedures that are probably not unnecessary to avoid possible legal consequences |  |  |  |
| Order tests that are probably not clinically indicated without a doctor's prescription to avoid possible legal consequences |  |  |  |
| Having severe concerns about making mistakes in nursing care |  |  |  |
| Explain nursing practices in more detail to protect yourself from malpractice allegations |  |  |  |
| Keep the records in a more detailed way to protect yourself from malpractice allegations |  |  |  |
| Refuse to assign high-risk patients to avoid possible legal consequences in the case of complications |  |  |  |
| Avoid high-risk procedures to avoid possible legal consequences in the case of complications |  |  |  |
| Administer drugs that you think to be unnecessary to protect yourself from malpractice allegations |  |  |  |
| Avoid patients who are more likely to file a lawsuit to protect yourself from malpractice allegations |  |  |  |
| Avoid practices with high complications to protect yourself from malpractice allegations |  |  |  |

### **Consent Form**

Title of the Study: **When Safety Becomes the Priority: Defensive Nursing Practice and its Associated Factors Among Nurses in Egypt: A Cross-Sectional Study**

we conducted this cross-sectional study to explore and analyze the prevalence and types of defensive nursing practices among nurses in Egypt. Also, identify the factors that contribute to the adoption of these practices.

**Please initial each box:**

[ ] I confirm that I have read and understand the information sheet dated [ ] for the above study.

[ ] I have had the opportunity to consider the information, ask questions, and have had these answered satisfactorily.

[ ] I understand that my participation is voluntary and that I am free to withdraw at any time, without giving any reason, without my medical care or legal rights being affected.

[ ] I agree to take part in the above study.

Name of Participant: ___________________________________

Signature of Participant: _______________________________

Date: ___________________

Name of Person Taking Consent: _________________________

Signature of Person Taking Consent: ______________________

Date: ___________________
